# Identification and characterization of a pathogenicity-related gene *VdCYP1* from *Verticillium dahliae*

**DOI:** 10.1038/srep27979

**Published:** 2016-06-22

**Authors:** Dan-Dan Zhang, Xin-Yan Wang, Jie-Yin Chen, Zhi-Qiang Kong, Yue-Jing Gui, Nan-Yang Li, Yu-Ming Bao, Xiao-Feng Dai

**Affiliations:** 1 The Institute of Food Science and Technology, Chinese Academy of Agricultural Sciences, Beijing 100193, P.R. China

## Abstract

*Verticillium dahliae* is a phytopathogenic fungus that causes vascular wilt disease in a wide variety of crop plants, thereby causing extensive economic loss. In present study, one *V. dahliae* T-DNA mutant M01C06 showed the pathogenicity loss on cotton, and the expression of a flanking gene encoding cytochrome P450 monooxygenase (P450, *VdCYP1*) was strongly repressed. P450s of fungi could affect the fungal pathogenicity by involving in the synthesis of secondary metabolites. However, there was no report about the pathogenic function of P450s in *V. dahliae*. *VdCYP1* gene deletion and complementation experiments confirmed that *VdCYP1* was the pathogenicity-related gene in *V. dahliae*. A comparison of culture supernatants of the *VdCYP1* deletion mutants and wild-type strains indicates that at least 14 kinds of secondary metabolites syntheses were affected due to *VdCYP1* gene deletion. One of these compounds, sulfacetamide, had the ability to induce the necrosis and wilting symptoms in cotton. Above results indicate that VdCYP1 could participate in pathogenesis by involving the secondary metabolism in *V. dahliae*, such as the compound sulfacetamide. In conclusion, *VdCYP1* acts as an important pathogenicity-related factor to involve in secondary metabolism that likely contributes to the pathogenic process in *V. dahliae.*

*Verticillium dahliae* is a soil-borne phytopathogenic fungus that causes destructive disease in over 200 plant species, including a wide range of economically important crops^1^,^2^. *V. dahliae* is a fungal genus in the *Ascomycota*, and it is an anamorphic form of the *Plectosphaerellaceae* family in the *Hypocreomycetidae* subclass of the *Sordariomycetes* class^3^. It could cause the serious Verticillium wilt on plants, and the symptom includes wilting, chlorosis, stunting, necrosis, vein clearing, and discoloration in stem tissue cross-sections^4^,^5^. Moreover, the dormant microsclerotia structures of *V. dahliae* remain viable in the soil for more than 20 years and cause serious Verticillium wilt symptoms after germination during plant infection^5^. Although *V. dahliae* has a clonal population structure with little or no evidence of recombination, the population is subdivided into multiple divergent lineages, and the extant lineage may have arisen by recombination from sexual ancestors^6^. Together, the Verticillium wilt causes by the *V. dahliae* is difficult to manage in an agricultural setting due to the broad host range, the notorious vascular system invading characteristic, the long-term survival of microsclerotia in soil, and unknown population divergence.

Recently, numerous genes involved in the pathogenicity have been identified in *V. dahliae*, such as *NLP1* and *NLP2* (encoding necrosis- and ethylene-inducing-like protein)^7^, *Ave1* (for avirulence on *Ve1* tomato)^8^, *VdSSP1* (encoding *V. dahliae*-specific secreted protein)^9^, *Vdsc1* (encoding isochorismatase)^10^, the *VdSge1* (encoding a transcriptional regulator)^11^, *Vta2* (encoding a zinc finger regulator)^12^, *VdSNF1* (encoding sucrose non-fermenting protein kinase)^13^, *VGB* (encoding G protein β subunit)^14^, *VdNUC-2* (encoding a *Neurospora crassa nuc-2* homolog)^15^, other pathogenicity-related genes *VDH1* (encoding the class II hydrophobin)^16^, and *VdTbi4* (encoding thiazole biosynthetic protein)^17^. All of these studies are beneficial for increasing the understanding of the biological properties of *V. dahliae*, and also strongly suggest that *V. dahliae* maybe employ numerous genes to contribute the pathogenic process.

*Agrobacterium tumefaciens*-mediated transformation (ATMT) has been particularly useful for the study of *V. dahliae* genes that are associated with diverse phenotypes^18^,^19^,^20^. Pathogenicity assays identified several mutants with reduced or lost pathogenicity, and the candidate genes were involved in *V. dahliae* pathogenicity. The genes included *VdHMGS* (encoding hydroxymethyglutaryl-CoA synthase), *VdEG-1* (encoding endoglucanase), *VdGPIM3* (encoding glycosylphosphatidylinositol mannosyltransferase 3), and *VdMFS1* (encoding major facilitator superfamily transporter)^18^; *VdGARP1* (encoding glutamic acid-rich protein)^19^; *VdPR3* (pathogenicity-related gene 3)^21^ and *VdCYC8* (encoding CYC8 glucose repression mediator protein)^22^. These results suggest that ATMT can be effectively used to identify genes that are associated with pathogenicity of *V. dahliae*. We previously used ATMT to perform T-DNA random insertional mutagenesis of Vd991, a highly virulent strain of *V. dahliae*, and a small mutant library of 2,628 mutants was obtained^20^.

To identify pathogenicity-related genes in *V. dahliae*, the virulence of 1,344 transformants was assayed on cotton plants, and 69 transformants were identified to display a significant reduction or loss of pathogenicity. One mutant strain, M01C06, the T-DNA insertion affected the expression of a candidate pathogenicity-related gene encoding a cytochrome P450 monooxygenase (named *VdCYP1*; VDAG_05890 in VdLs.17 genome), resulting in a near-complete loss of pathogenicity on cotton plants. P450s are heme-thiolate proteins distributed across the biological kingdoms, and involved in the metabolism of a diverse array of endogenous and xenobiotic compounds^23^,^24^,^25^. Especially, P450s extensively participate in a wide variety of physiological reactions in fungi that contribute to the fitness and fecundity of fungi in various ecological niches^26^. Analysis of fungal genomes has revealed the presence of a surprisingly large number of P450s which often involve in oxygenation steps in the biosynthesis of fungal secondary metabolites^23^,^27^,^28^. Moreover, the P450 family shows significantly expansion in several plant pathogens by the comparative genomics analysis^23^,^25^, and numerous P450s are significantly regulated during host-pathogen interactions to involve in fungal pathogenic processes^29^,^30^, such as in *Heterobasidion annosum* sensu lato^31^, *Moniliophthora perniciosa*^32^, *Botrytis cinerea*^33^, and *Fusarium spp.*^34^. Taken together, the previous reports suggested that the P450s probably involve in the secondary metabolism and plays an important role in the pathogenesis. However, the involvement of P450s in *V. dahliae* pathogenicity has not been reported.

In this study, we mainly focused on determining whether *VdCYP1* is a pathogenicity-related gene in *V. dahliae*. The expression of *VdCYP1* was disturbed by the T-DNA insertion in the M01C06 mutant, which showed a loss of virulence on the cotton plant. Further gene deletion and complementary experiments confirmed that gene *VdCYP1* contribute to virulence during infection. And the secondary metabolites analysis indicates that *VdCYP1* may be involved in the secondary metabolism, by which to affect the pathogenesis on cotton.

## Results

### Identification of a non-pathogenic mutant of *V. dahliae*

**Figure 1:**
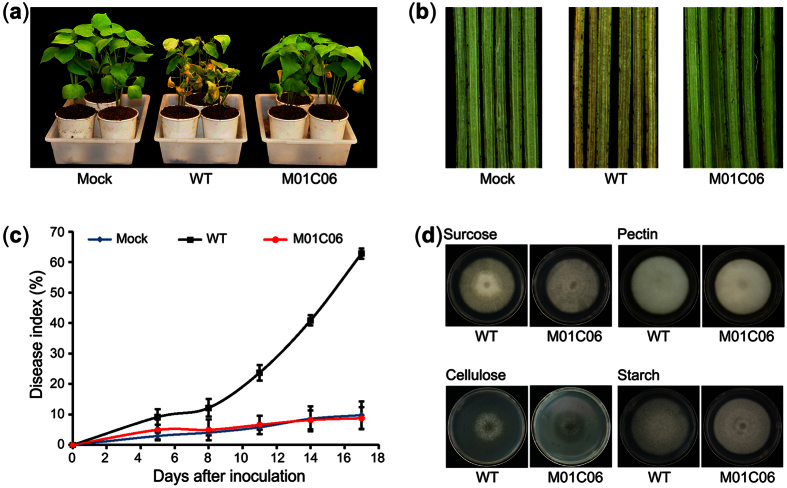
Detection of the pathogenicity and growth phenotypes of the M01C06 mutant. (**a**) The pathogenicity of the M01C06 mutant on cotton. The pathogenicity was detected using the root-dipping method (see Materials and methods). Images were taken 17 days after inoculation. WT shows the wild-type strain Vd991, and Mock indicates the control that was treated with sterile water. (**b**) Investigation of vascular discoloration after infection with M01C06. Vertical sections of cotton plants were photographed 17 days after inoculation. (**c**) Assessment of the pathogenicity of M01C06 using the DI. The degree of Verticillium wilt was determined by statistical analysis using the DI method (see Materials and methods). (**d**) The growth phenotypes of M01C06 on media containing the carbon sources sucrose, pectin, cellulose, and starch. Images were captured after 11 days of incubation at 25 °C.

Using the root-dipping method with susceptible cotton (*Gossypium hirsutum* cv. Junmian 1), mutants showing changes in virulence were isolated from a small T-DNA random insertion library that contained 1,344 mutants. The library was generated from a highly aggressive defoliating strain of *V. dahliae*, Vd991, by using ATMT. In total, 69 mutants exhibited reduced virulence relative to the wild-type strain, and 16 of them showed significantly less pathogenicity to cotton (see Supplementary Fig. S1). Further assessment of their pathogenicity on cotton plants confirmed that one of the mutants, termed M01C06, showed near-complete loss pathogenicity to cotton, while the wild-type Vd991 strain caused severe necrosis and wilting of the leaves (Fig. 1a). Investigation of vertical shoot sections showed that the M01C06 mutant almost lost all capacity for vascular discoloration when compared to the symptoms associated with wild-type infection (Fig. 1b). The disease index (DI) of cotton inoculated with the M01C06 mutant was significantly lower than that of the wild-type strain. The M01C06 DI was 8.78 ± 3.07, similar to the value for mock-inoculation (which is caused by cotyledon senescence under normal conditions), while the wild-type Vd991 strain showed a DI of 68.05 ± 3.62 after inoculation (Fig. 1c).

The colony-growth phenotype of the M01C06 mutant was unchanged relative to wild-type strains. The development rates and colony diameters were similar on media containing several different carbon sources, including sucrose, pectin, cellulose, and starch (Fig. 1d). Together, these results revealed that the M01C06 mutant showed a significantly reduced virulence on cotton but did not present alterations in development, implying that the gene associated with the integration of T-DNA in the genome was specifically involved in the pathogenicity of *V. dahliae*.

### Analysis the T-DNA tagged genes in the mutant

**Figure 2:**
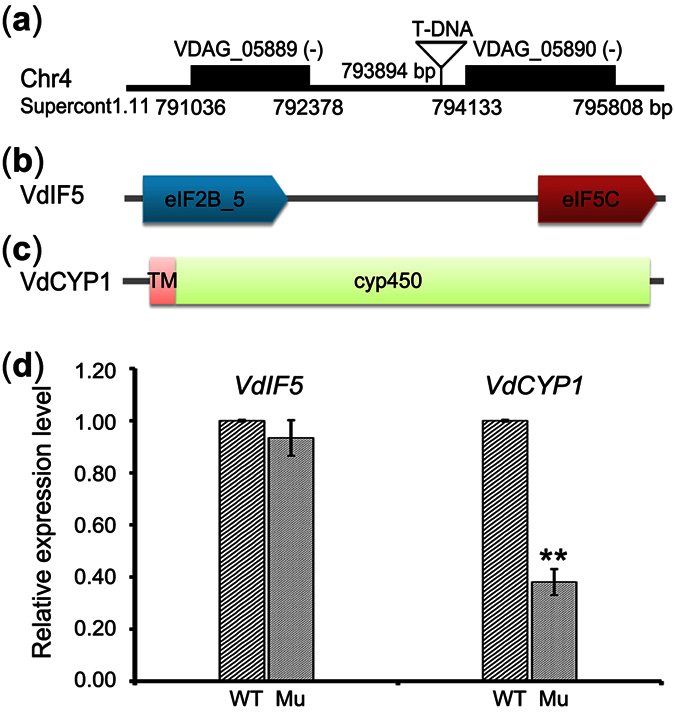
Analysis of T-DNA tagged genes in a mutant strain. (**a**) The physical location of the T-DNA insertion into the intergenic region in the mutant; the minus sign in the bracket represents the gene encoded in the antisense orientation. (**b,c**) Prediction of the conserved domains in *VdIF1* and *VdCYP1*; the domains were predicted using SMART (http://smart.embl-heidelberg.de/). The eIF2B_5 gene encodes eukaryotic initiation factor 2B_5, eIF5C is eukaryotic initiation factor 5C, TM represents the transmembrane segment, and cyp450 indicates cytochrome P450. (**d**) Detection of the relative expression levels of *VdIF5* and *VdCYP1* in mutant and wild-type strains. WT and Mu represent the wild-type and mutant strain M01C06, respectively. Error bars are standard error, **indicate significant differences (*P* < 0.01), unpaired Student’s *t*-test.

To identify the targeted gene that was involved in the pathogenicity of M01C06 mutant, the sequences flanking the T-DNA insertion were isolated from the mutant using hiTAIL-PCR as previously described^35^. Fragments of approximately 200–1000 bp were amplified from the genomic DNA of the mutant using 5 degenerate primers (see Supplementary Fig. S2). Among the amplified fragments, most were impaired PCR amplicons, with the exception of a 795-bp amplicon that completely matched the T-DNA and its flanking sequence; the sequence indicated that the T-DNA integrated into an intergenic region at nucleotide 793,894 of supercontig 1.11 (chromosome 4) between the VDAG_05889 and VDAG_05890 genes, according to the reference genome for VdLs.17^36^. The physical distances of *VdIF5* and *VdCYP1* from the T-DNA insertion were 1,471 bp and 286 bp, respectively (Fig. 2a). VDAG_05889 (termed *VdIF5*) encodes a protein that is homologous to eukaryotic translation initiation factor 5 (Fig. 2b), which plays a central role in protein synthesis by organizing the formation of the pre-initiation complex^37^. VDAG_05890 (termed *VdCYP1*) is predicted to encode a cytochrome P450 monooxygenase belonging to the P450s superfamily (Fig. 2c), the proteins of which are involved in many essential cellular processes and play diverse functional in fungi^28^. A transcript-level comparison of *VdIF5* and *VdCYP1* in the mutant showed that the relative expression of *VdCYP1* but not *VdIF5* was markedly reduced relative to that in the wild-type strain (to only 38% of the level in the wild-type strain; Fig. 2d), indicating that the integration of the T-DNA into the intergenic region likely affects the function of *VdCYP1* in the M01C06 mutant.

### Identification of the cytochrome P450 monooxygenase-encoding *VdCYP1* gene

**Figure 3:**
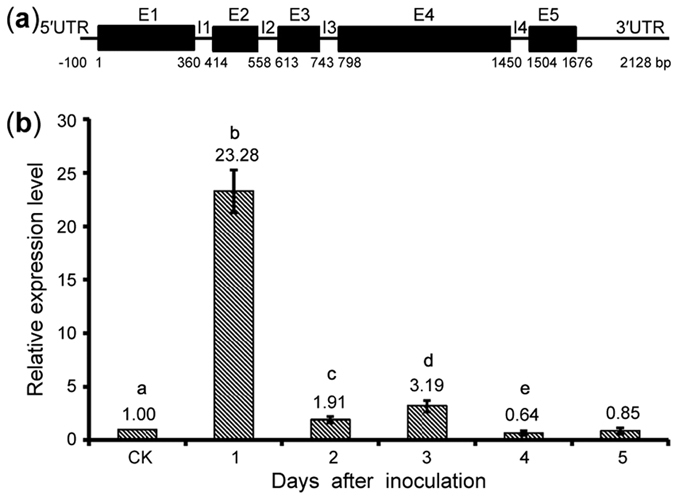
The gene structure of *VdCYP1* and its expression changes in response to cotton. (**a**) The gene structure of *VdCYP1*. E1–E5 and I1–I4 indicate the five exons and four introns, respectively. (**b**) Changes in the expression of *VdCYP1* during the *V. dahliae* response to cotton. CK represents hyphae collected from Czapek-Dox medium and the expression of *VdCYP1* was set to 1.Different letters represent significant difference (*P* < 0.05), unpaired Student’s *t*-test.

Based on information predicting that the VDAG_05890 gene contains 4 introns in VdLs.17 genome^36^, the *VdCYP1* transcript was obtained using RT-PCR (see Supplementary Fig. S3a). The sequence of the cDNA verified that the full-length open reading frame was 1,458 bp in length and encoded 485 amino acids (Fig. 3a). After comparison, the genomic sequence of *VdCYP1* was confirmed to contain four introns (Fig. 3a). Therefore, the *VdCYP1* gene had the identical sequence and gene structure as the annotation in VdLs.17 genome^36^. Moreover, a 506-bp 3′ untranslated region (3′ UTR) was cloned using 3′ rapid amplification of cDNA ends; this region included the integration site of the T-DNA (nucleotide 793,894 of supercontig 1.11 in VdLs.17) (see Supplementary Fig. S3b,c). These results confirmed that the T-DNA was integrated into the 3′ UTR of *VdCYP1*, providing an explanation for the suppressed expression of the *VdCYP1* gene that ultimately affected the pathogenicity of the M01C06 mutant.

Bioinformatics analysis of the VdCYP1 protein confirmed that VdCYP1 contains a P450 domain (IPR002401). Further comparison to the Fungal Cytochrome P450 Database^23^ revealed that VdCYP1 had a greater similarity to members of group I of the E-class P450 proteins (CYP family 548), which includes numerous orthologous genes in fungi. Although there is high variation in the amino acid sequences of VdCYP1 and its orthologs, group I of the E-class P450 family contains six conserved signature motifs (E-I), four of which are conserved in VdCYP1 (see Supplementary Fig. S4). In particular, P450s contain the highly conserved cysteine heme-iron ligand signature at the C-terminal end of the protein^24^. Correspondingly, VdCYP1 encodes the signature sequence of ten amino acids (FAFANGGRGC) in the E-I-5 motif, including a conserved cysteine residue (see Supplementary Fig. S4).

Several P450s have been implicated in mechanisms of plant pathogenicity^28^. To determine whether *VdCYP1* is related to *V. dahliae* pathogenicity, the expression level of *VdCYP1* was detected during the cotton infection process using quantitative real-time PCR. As expected, the expression of *VdCYP1* was continuously up-regulated within five days after infection of cotton; the transcript level was significantly elevated by over 20-fold one day after inoculation (Fig. 3b). These results suggest that *VdCYP1* may be involved in *V. dahliae* pathogenicity and that it plays a curial role in the extreme environment of the cotton cell wall during the early infection process.

### *VdCYP1* is crucial for the virulence of *V. dahliae*

**Figure 4:**
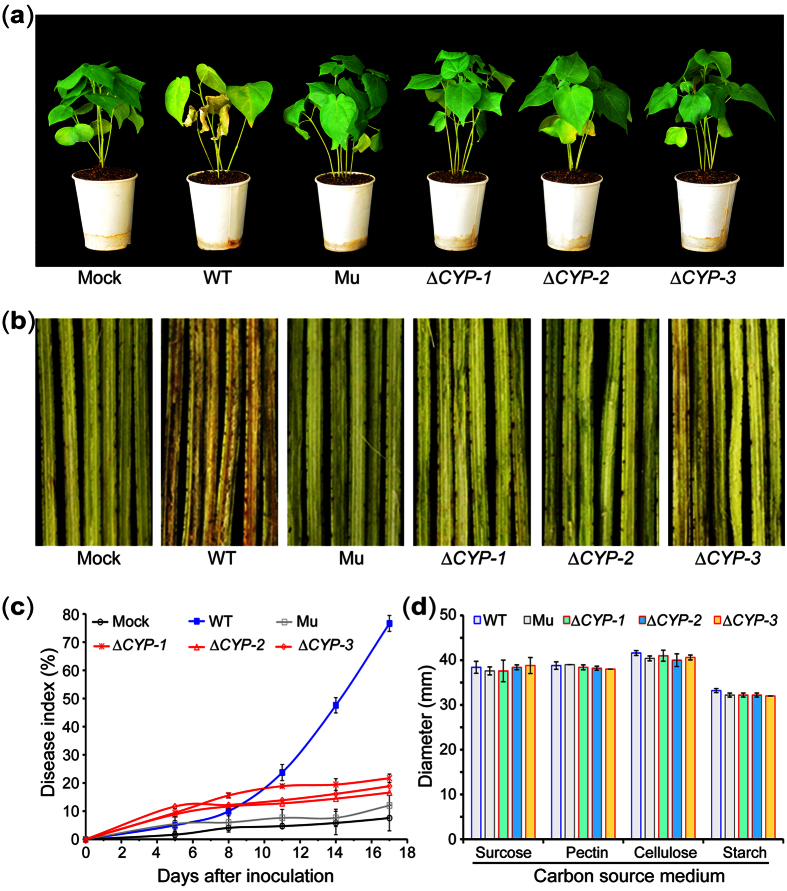
The pathogenicity and growth phenotypes of *VdCYP1* deletion strains. (**a**) The pathogenicity phenotypes of *VdCYP1* deletion strains with respect to cotton. Shown are three *VdCYP1* deletion strains (Δ*CYP*-1-3), the T-DNA insertion mutant M01C06 (Mu), and the Vd991 wild-type strain (WT); the mock inoculation was treated with sterile water. (**b**) The stem discoloration induced by infection of cotton with the *VdCYP1* deletion strains. (**c**) The DI of Verticillium wilt after infection with the three *VdCYP1* deletion strains. (**d**) The radial growth of the three *VdCYP1* deletion strains. The strains were cultured on media containing sucrose, pectin, cellulose or starch as carbon sources.

To determine whether *VdCYP1* was the disrupted gene that caused the loss of pathogenicity in M01C06 mutant, knockout transformants of *VdCYP1* and *VdIF5* were generated in the Vd991 wild-type strain by using homologous recombination with ATMT (see Supplementary Fig. S5). As expected, the *VdCYP1* gene was necessary for the virulence of the wild-type strain. The pathogenicity of three random knockout strains (Δ*VdCYP1*-1-3) was significantly reduced on cotton, and these strains caused no aberrant stunting, chlorosis or wilting symptoms in the cotton plants, similar to the phenotype observed after inoculation with the T-DNA M01C06 mutant (Fig. 4a). Moreover, deletion of *VdCYP1* attenuated vascular discoloration in cotton in a way that was observably different from the wild-type strain (Fig. 4b). The degree of cotton leaf wilting that was observed with the knockout strains was also markedly reduced compared to that of the wild-type strain, and the knockout strains showed DI values of only 16.22 ± 4.08 after 17 days inoculation, similar to the value of the T-DNA mutant M01C06 (Fig. 4c). In addition, the phenotypes of the *VdCYP1* deletion strains and their radial growth on media with different carbon sources were similar to those of the wild-type strain during the development process over 11 days, with the exception of a minor difference in hyphal compaction (Fig. 4d; see Supplementary Fig. S6). As expected, the homologous recombination of *VdIF5* was unsuccessful after ATMT in five experiments with six replicates (see Supplementary Fig. S5c), which probably due to the function of *VdIF5* should be necessary for basic biological processes involving the eukaryotic translation initiation factor, such as protein translation. These results, together with our observation in mutant M01C06, show that the integration of T-DNA in the intergenic area significantly affected the function of *VdCYP1* but not *VdIF5* and caused a loss of pathogenicity on cotton, strongly indicate that gene *VdCYP1* is crucial for the virulence of *V. dahliae*.

### Re-introduction of *VdCYP1* into the T-DNA mutant and knockout strains restores the pathogenicity on cotton

**Figure 5:**
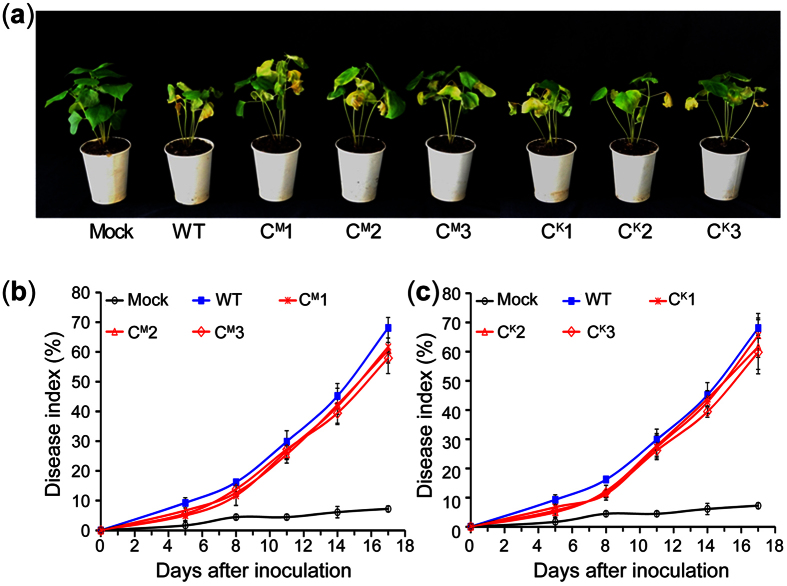
The pathogenicity of the T-DNA mutant and knockout strains on cotton after complementation with *VdCYP1*. (**a**) The pathogenicity of transformants with a re-introduced *VdCYP1* gene. (**b**,**c**) The degree of Verticillium wilt after infection with the complemented transformants. Three complemented transformants were selected for each pathogenicity analysis; C^Mu^-1, C^Mu^-2 and C^Mu^-3 represent strains with *VdCYP1* re-introduced into the M01C06 mutant; C^KO^-1, C^KO^-2 and C^KO^-3 represent strains with *VdCYP1* re-introduced into the deletion strain Δ*VdCYP1*. WT represents the wild-type strain Vd991, and Mock represents the control that was treated with sterile water.

To ensure that the pathogenicity loss in the *VdCYP1* deletion strain was caused by the T-DNA insertion or the gene replacement event, the full-length of gene *VdCYP1* was re-introduced into the T-DNA mutant and knockout strains using ATMT with geneticin selection. The transformants in which whether the *VdCYP1* gene has been integrated were verified by PCR. Three complementation transformants, C^M^1, C^M^2, and C^M^3, in which the *VdCYP1* gene was integrated in the T-DNA M01C06 mutant background, and three other transformants, C^K^1, C^K^2, and C^K^3, in which the *VdCYP1* gene was integrated in the gene knockout strains, were selected for phenotypic analysis (see Supplementary Fig. S7). As expected, the virulence of above six transformants were restored on cotton after re-introducing the *VdCYP1* gene into the gene deletion and T-DNA strains, showing the virulence comparable to that of the wild-type Vd991 strain (Fig. 5a). Above results confirm that *VdCYP1* acts as a pathogenic gene in *V. dahliae*. An assay of the degree of Verticillium wilt symptoms showed that the DI values of all the transformants were between 57.95 ± 1.67 and 65.56 ± 7.52, which were close to that of the wild-type strain (approximately 68.0). These results reconfirmed that *VdCYP1* acts as a pathogenic factor and is crucial for the virulence of *V. dahliae*.

### VdCYP1 involved in the secondary metabolism in *V. dahliae*

Previous research on fungal genomes revealed that the genome encodes a large number of P450 genes, which are typically involved in fungal pathogenic processes by participating in the biosynthesis of fungal secondary metabolites^23^,^27^,^28^. To determine whether *VdCYP1* is involved in *V. dahliae* secondary metabolism, extracellular metabolites were extracted from the culture supernatants of two independent knockout mutant strains (Δ*VdCYP1*-1 and Δ*VdCYP1*-2) and the Vd991 wild-type strain. The metabolites were detected using an ultra-performance liquid chromatography-quadrupole time-of-flight mass spectrometry (UPLC-Q-TOF-MS) method and many compounds showed significant difference between wild-type strain and *VdCYP1* deletion mutants (see Supplementary Table S1). Unexpectedly, the qualitative analysis found that the number of the different compounds presented significant difference in the two independent knockout mutants compared with the wild-type strain, identified that 206 and 699 compounds in the Δ*VdCYP1*-1 and Δ*VdCYP1*-2 supernatants, respectively, were less than in the wild type (see Supplementary Table S1). These results suggest that the secondary metabolism was extremely sensitive to the independent recombination events of *VdCYP1* in *V. dahliae*. Further combination analysis found that the metabolism of 14 compounds (Sulfacetamide, 1-Methoxy-1H-indole-3-acetonitrile, Sinalexin, etc.) was simultaneously affected both in the Δ*VdCYP1*-1 and Δ*VdCYP1*-2 mutants compared to the wild-type strain (Table 1). This result provided the solid evidence for the opinion that VdCYP1 could be involved in the secondary metabolism of *V. dahliae*.

**Figure 6:**
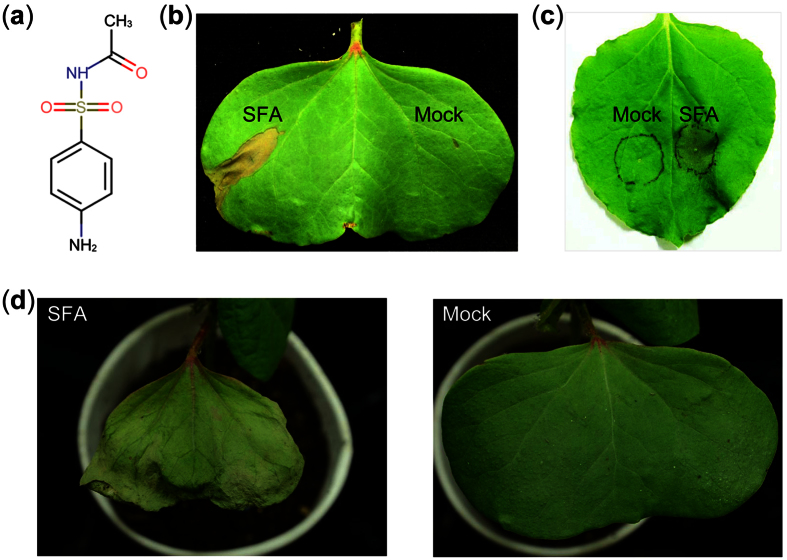
The wilting activity of sulfacetamide metabolites. (**a**) The chemical formula of sulfacetamide. (**b**) The wilting activity on cotton leaves following infiltration with sulfacetamide (SFA) solution (25 mM); the mock-treated leaves were infiltrated with the 2% DMSO water solution. The phenotype was photographed 24 hours after infiltration. (**c**) Detection of the wilting activity in tobacco leaves after infiltration with the SFA solution (25 mM). (**d**) Validation of the wilting activity of cotton plants *in vivo* using the root-dipping method. Cotton seedlings with second euphylla were treated with a 25 mM SFA solution, while the mock-treated seedlings were treated with 2% DMSO water solution. The phenotype was photographed 36 hours after inoculation.

According to the KEGG annotation of the reference genome of VdLs.17^36^, at least 148 genes are involved in aminobenzoate degradation pathway, and among them 41 genes encode proteins containing the P450 domain (see Supplementary Table S2). In particular, the pathway annotation of *VdCYP1* was matched to the reaction of parathion to paraoxon by oxygenation (see Supplementary Fig. S8), indicating that *VdCYP1* may be involved in the aminobenzoate degradation pathway. However, we did not find the variation of parathion or paraoxon in Δ*VdCYP1*-1 and Δ*VdCYP1*-2 strains, which indicate that parathion or paraoxon was not the final products of VdCYP1 in the aminobenzoate degradation pathway. Interestingly, the result of UPLC-Q-TOF-MS showed that the amount of one typical compound, sulfacetamide (SFA), was significantly higher in the wild-type strain than in the two independent knockout mutants (Table 1). Coincidentally, the structure of sulfacetamide is similar to sulfanilic acid, which is one metabolite of the aminobenzoate degradation pathway. Therefore, we infer that gene *VdCYP1* probably affect the metabolism of the sulfacetamide by involving aminobenzoate degradation pathway. In addition, previous reports indicated that sulfacetamide derivatives are toxic compounds that can inhibit the biosynthetic pathway of folate, which is an essential molecule that is required by numerous organisms, especially plants^38^,^39^,^40^. Correspondingly, the *in vitro* assay showed that the cotton and tobacco leaves were sensitive to sulfacetamide, and the cotton and tobacco leaves all showed the obvious necrosis and wilting symptoms after infiltration with the sulfacetamide solution (Fig. 6b,c). Moreover, *in vivo* detection also showed that sulfacetamide could cause significant wilting symptom in cotton leaves that was similar to the typical disease symptoms (darkening and wilting of the leaves) of Verticillium wilt (Fig. 6d). Therefore, above results indicate that *VdCYP1* play important function in the pathogenesis of *V. dahliae* by involving in the secondary metabolism, such as the aminobenzoate degradation pathway, to contribute to virulence during infection.

## Discussion

Recently, several studies of *V. dahliae* have revealed that this pathogen could employ various and complex mechanisms for penetration, colonization, host-pathogen interaction, and expansion in plants; and the complex mechanisms include the utilization of carbon/nitrogen metabolism to obtain nutrition, overcoming the osmotic stress and reactive oxidative species produced by the host plant in response to infection, and modulating host physiology or biochemistry with effectors^41^. Correspondingly, numerous genes have been identified as pathogenicity-related factors in *V. dahliae* and facilitate the pathogenic progression, including the cell wall-degrading enzymes^9^,^18^, various effectors^7^,^10^,^11^,^42^, protein kinases^13^, and transcriptional regulators^11^,^12^. With the completion of the genome sequence of *V. dahliae*^36^,^42^, ATMT technology has been more widely used for the study of *V. dahliae* genes that are associated with diverse phenotypes^18^,^19^,^20^, especially the genes associated with pathogenicity. At present, several genes associated with pathogenicity have been identified from the mutants with reduced or lost pathogenicity^18^,^19^,^21^,^22^, confirming that ATMT is an effective method to identify the genes associated with *V. dahliae* pathogenicity.

In China, Verticillium wilt caused by *V. dahliae* is the most destructive disease of cotton, as more than 50% of the cotton acreage has been affected by Verticillium wilt in recent years, significantly reducing the fiber quality and resulting in yield losses (National Cotton Council of America Disease Database). Based on the premise that the resistant genes are lacking in cotton, the investigations of pathogenicity-related genes and revealing the pathogenic mechanism of *V. dahliae* could be beneficial for the development of Verticillium wilt control strategies. In our previous research, the T-DNA random insertional mutagenesis of Vd991 (the typical highly virulent strain of the cotton host origin) was obtained using ATMT, which prompted us to investigate the pathogenicity of *V. dahliae*^20^. In present research, at least 16 mutants with significant pathogenicity reduce or loss were screened (see Supplementary Fig. S1). And the locations of the T-DNA insertion sites have already revealed several genes associated with pathogenicity phenotypes, such as *VdCYP1* (encoding the cytochrome P450 monooxygenase), *VdPT1* (encoding the palmitoyltransferase), *VdVSP1* (encoding the vacuolar protein sorting-associated protein), and *VdOR1* (encoding the oxidoreductase) (unpublished data). These results confirmed that the ATMT methods can be effectively used in the study of pathogenicity-related genes in *V. dahliae*.

In present research, one T-DNA mutant that lost the pathogenicity on cotton was selected for pathogenesis analysis (Fig. 1). The identification of the flanking sequence indicate that the T-DNA was integrated into the intergenic region between gene *VdCYP1* and *VdIF5* (Fig. 2); and the further targeted gene deletion and complement confirmed that *VdCYP1* was the pathogenic related gene (Figs 4 and 5). The protein encoded by *VdCYP1* is highly similar to P450s of other fungi, and it contains the conserved region with the sequence FXXGXXXCXG (see Supplementary Fig. S4), which is the heme-binding domain that contains the axial Cys ligand for the heme^24^. P450s as a superfamily of heme-containing monooxygenases, distribute widely in fungi, and play diverse and pivotal roles in metabolic versatility and fungal adaptation to specific ecological niches^23^,^25^. Associations between P450s and pathogenicity have been identified in many fungi^30^,^33^,^43^,^44^, and several studies showed that mycotoxin synthesis in several fungi requires the participation of P450s in the pathogenic process^45^. Moreover, comparative genomics revealed that P450s family in several plant pathogens has been significantly expanded to promote the pathogenicity and adaptation^23^,^25^. Numerous P450s have been found to be significantly up-regulated during host-pathogen interactions^29^,^30^; such regulation also occurred in *V. dahliae*, as *VdCYP1* was observably up-regulated during cotton infection (Fig. 3). Together, our research strongly indicates that *VdCYP1*, which encodes a P450, is a pathogenicity-related gene to involve in the pathogenicity in *V. dahliae*.

Secondary metabolites can be the pathogenic or virulent factors and cause significant disease in agricultural crops^46^, as demonstrated by several studies of *Fusarium spp*^34^. For example, the trichothecene mycotoxin that are produced by certain *Fusarium* species can promote the virulence towards wheat and maize^47^,^48^, by inhibiting protein expression and elicitor-inducing activities that could stimulate the plant defenses and promote plant cell death^49^. The increasing evidences suggest that P450s, as one huge and incompletely discovered family, could play important and various roles in the pathogenic processes of fungi infecting host plants by participating in the secondary metabolites synthesis^44^. Recent fungal transcriptome data analysis found that the expression of many fungi P450 genes have been obviously induced in infection process, which maybe influence the fungal secondary metabolites synthesis and participate in the pathogenic process. For example, one putative benzoate 4-monooxygenase cytochrome P450 gene of *Fusarium graminearum* was obviously induced during wheat coleoptiles infection, and was speculate to involve in the pathogenic process by participating in the secondary metabolism^29^. The idea that P450s could involve in secondary metabolites synthesis and influence fungi pathogenicity was also confirmed in our investigation, as the qualitative analysis of some secondary metabolites showed observably decrease in Δ*VdCYP1* mutants compared to the wild-type strain (see Supplementary Table S1). In particular, 14 compounds were thought to be affected by the *VdCYP1* deletion in the two independent Δ*VdCYP1* mutants (Table 1). But until now, there were no other report about the regulatory relationship between P450 and these compounds, and the possible pathogenic function of these compounds were also unclear.

The KEGG annotation found that VdCPY1 might be involved in the aminobenzoate degradation pathway (see Supplementary Fig. S8 and Table S2). Coincidentally, one of the 14 kinds of compounds, sulfacetamide, which is the analogous compound of sulfanilic acid that involves in the aminobenzoate degradation pathway, could cause the necrosis and wilting symptom on cotton showed by the *in vivo* and *vitro* assay (Fig. 6). These result indicate that VdCPY1 may be involved in the aminobenzoate degradation pathway of *V. dahliae* and indirectly influence the synthesis of sulfacetamide, which could act as one virulent factor during infecting cotton. This study is the first report of the characterization and pathogenic function analysis of one P450 gene *VdCYP1* in *V. dahliae*, and this gene could affect the pathogenicity of *V. dahliae* by influencing the synthesis of secondary metabolites synthesis, such as the metabolism of sulfacetamide, which probably one of the typical case in its’ complex secondary metabolic regulatory functions.

Above results suggest that compound sulfacetamide probably played as one virulent factor in *V. dahliae* pathogenic process. Still, the pathogenic mechanism of the compound sulfacetamide in cotton was also unclear. Previous reports indicate that sulfacetamide can impair folate synthesis in *Arabidopsis thaliana*^50^. And folate as essential water-soluble B-vitamins is required by numerous organisms, especially on the plants^51^. During folate synthesis, dihydropteroate synthase (DHPS) acts as one of the crucial enzymes that catalyzes the condensation of dihydropteridine diphosphate and para-aminobenzoate to yield dihydropteroate^52^,^53^. Sulfonamide is an analogue of para-aminobenzoate and can act as a competitive inhibitor of DHPS^53^ that can inhibit DHPS activity, and finally disturb the folate synthesis in *Arabidopsis*^51^. Therefore, VdCYP1 may involve in the secondary metabolism by the aminobenzoate degradation pathway to influence the compounds sulfacetamide that could impair folate synthesis and ultimately induce the wilting symptom in host plants.

In conclusion, P450 VdCYP1 is one necessary pathogenic factor during *V. dahliae* infecting cotton. VdCYP1 could involve in the synthesis of secondary metabolites in *V. dahliae*, such as the pathogenic compound sulfacetamide synthesis. The characterization of *VdCYP1* expression and pathogenic function during *V. dahliae* infecting cotton in this study would provide insight into the process of *V. dahliae* infecting host plant, promote the identification of P450s’ roles in *V. dahliae* secondary metabolism and accelerate the understanding of other functions of P450s in fungal pathogenic process.

## Materials and Methods

### Identification of mutants with a loss of pathogenicity

The T-DNA library, consisting of 1,344 mutants, was cultured on potato dextrose agar (PDA) medium at 25 °C for 10 days before the conidia were washed with sterile water and the concentration was adjusted to 5 × 10^6^ conidia/mL. The susceptible cotton (*G. hisutum* cv. Junmian 1) was planted in sterilized soil at 28 °C with a 16/8 light-dark photoperiod for 2 weeks. In the preliminary screening, 12 cotton seedlings were immersed into 30 mL of prepared conidial suspension for 2 minutes and then transferred into a new nutrition pot before being cultured in the greenhouse at 28 °C with a 16/8 light-dark photoperiod for 3 weeks. The pathogenic loss mutants were further selected using a pathogenicity assay with an expanded population of 30 cotton seedlings with 3 replicates. Symptom development was measured from the first day to the sixth week and categorized according to the disease index (DI), which uses four grades: grade 1: the cotyledon beginning to yellow; grade 2: the cotyledon and one euphylla wilting; grade 3: all leaves wilting and chlorosis; and grade 4: plant death. The formula of DI value = [∑ (the seedling of every grade × relative grade)/(total seedlings × 4)] × 100^54^. The symptoms of Verticillium wilt were photographed after three weeks inoculation. The pathogenicity loss mutant M01C06 was further used for functional analysis.

### T-DNA tagged gene analysis in the mutant

The M01C06 mutant was cultured on liquid complete medium (CM) at 25 °C for 5 days, and the mycelium was then harvested for genomic DNA isolation using the DNAsecure Plant Kit (TianGen Biotech., CO., Ltd., Beijing, China). The flanking sequences of T-DNA were identified using hiTAIL-PCR methods with degenerate primers, as previously described^35^. All amplicons were cloned, sequenced, and then aligned to the reference genome of VdLs.17 using BLASTN (http://blast.ncbi.nlm.nih.gov/Blast.cgi) to ascertain the physical location of the T-DNA. According to the alignment results, the 2 sequences flanking the T-DNA were reconfirmed using PCR with specific primers. Quantitative reverse transcription-PCR (qRT-PCR) was performed to identify the expression levels of genes that were affected by the T-DNA insertion using the FastFire qPCR premix (SYBR Green, TianGen, Beijing, China), and the relative gene expression level was calculated using the 2^−△△CT^ method^55^ with the β-tubulin gene (VDAG_10074, VdLs.17) as an internal control. The primers are listed in Supplementary Table S3. Real-time PCR conditions consisted of an initial 94 °C denaturation step for 10 min that was followed by 40 cycles of 94 °C for 15 s and 60 °C for 1 min.

### Gene cloning and bioinformatic analysis

Total RNA was extracted using the Total RNA Miniprep Kit (Axygen, MA, USA), and first-strand cDNA was synthesized using the FastQuant cDNA Reverse Transcriptase Kit (TianGen, Beijing, China). The flanking genes were cloned using RT-PCR with cDNA and PCR with genomic DNA, and then the protein-coding genes were identified using Clustal X software with the transcript and genomic sequence information (http://www.clustal.org/clustal2/). The rapid amplification of cDNA ends (RACE) system (Invitrogen, Carlsbad, California, USA) was used for 3′ end amplification using the standard protocol. Conserved domain and functional homology searches were performed using the SMART programs (http://smart.embl-heidelberg.de/smart). All primers are listed in Supplementary Table S3.

### Fungal transformation

The homologous recombination method was used to generate deletion mutants of the two flanking genes, *VdIF5* and *VdCYP1*, as previously described^9^. The flanking sequences were amplified from the genomic DNA, integrated into a hygromycin cassette using fusion PCR, and then assembled into the pGKO2-gateway vector^56^ in a BP recombinant reaction (Invitrogen). To generate the complementation vector, 3 fragments of the trpC promoter, open reading frame of *VdCYP1* and Nos terminator were fused using fusion PCR to obtain a complementation fragment with *Kpn*I/*Xba*I enzyme sites. The fragment was then ligated into the pCT-HN vector, which possesses hygromycin and geneticin resistance selection markers^9^.

The vectors were transferred into the *Agrobacterium tumefaciens* AGL-1 strain for fungal transformation. *A. tumefaciens*-mediated transformation of *V. dahliae* was conducted for the homologous recombination and complementation transformants as described previously^57^, and transformants were selected on PDA medium supplemented with hygromycin or geneticin at 50 μg/mL. Homologous recombination was verified by PCR. The primer sequences are shown in Supplementary Table S3.

### Gene expression analysis

To determine the expression level of *VdCYP1* during cotton infection, two-week-old cotton seedlings were root-inoculated with 5 × 10^6^ conidia/mL of a *V. dahliae* suspension; the roots were then harvested at 1, 2, 3, 4, and 5 days post-inoculation and flash frozen in liquid nitrogen for RNA extraction. *V. dahliae* cultured on PDA medium without being used to inoculate cotton plants was set as the control. After grinding, 100 mg of ground material was used for total RNA extraction and cDNA synthesis. The transcript level of *VdCYP1* was determined using qPCR, as described above.

### Phenotypic characterization

To determine the colony morphology on different carbon sources, conidial suspensions of *V. dahliae* strains were prepared at 2 × 10^6^ conidia/mL and placed in the center of basic Czapek-Dox medium agar plates that included either sucrose (30.0 g/L), pectin (10.0 g/L), cellulose (10.0 g/L) or starch (15.0 g/L). The growth phenotypes were investigated after 10 days of incubation at 25 °C, and the colony diameter was measured. Pathogenicity assays of the deletion strains and the complemented transformants were conducted with 30 cotton seedlings and in triplicate. The severity of Verticillium wilt was calculated using the DI method, as used to quantify the pathogenicity of T-DNA mutants. Phenotypic investigations of Verticillium wilt in whole plants and in vertical sections of the cotton seedling shoots were also conducted at three weeks post-inoculation.

### Secondary metabolite determination

The secondary metabolites in the culture supernatants of the *VdCYP1* deletion strains were identified using the UPLC-Q-TOF method. Two independent *VdCYP1* deletion strains were cultured in basic Czapek-Dox medium without antibiotics at 25 °C in a shaking incubator at 150 rpm for five days. The cultured solution was immediately centrifuged at 15,000 g at 4 °C for 20 min to collect the supernatants. The supernatants were filtered through a cellulose acetate filter (0.45 μm) and then cleaned up by liquid-liquid extraction (LLE) using 250 mL of chloroform:acetonitrile (1:1 v/v) twice. Next, the lower layer (organic solvent) was collected in a round-bottom flask and concentrated to near-dryness using a rotary evaporator at 35 °C. The residue was reconstituted in 2.0 mL of acetonitrile:water (50:50 v/v, containing 0.1% formic acid) and was then briefly centrifuged. Subsequently, 1.0 mL of the upper layer (acetonitrile) was filtered through a 0.22-μm syringe filter for UPLC-Q-TOF analysis. The wild-type strain was used as the control.

Samples were analyzed on an UPLC-Q-TOF mass spectrometer (Waters, Manchester, U.K.). Metabolites were separated using a Waters BEH Shield RP C18 column (50 mm × 2.1 mm, 1.7 μm) at 30 °C. The mobile phases were composed of phase A (0.1% formic acid in water) and phase B (0.1% formic acid in acetonitrile). The gradient conditions were optimized as follows: 0–1 min, 2–5% B; 1–8 min, 5–90% B; 8–10 min, 90–100% B; 10–12 min, 100–2% B. The flow rate was 0.3 mL/min. The MS system was operated in ESI+ mode. The mass range was set at m/z 100–1000 Da in the full-scan mode. The optimized ESI parameters were as follows: capillary voltage, 3.0 kV; cone voltage, 35 V; source temperature, 100 °C; desolvation temperature, 350 °C; desolvation gas flow, 600 L/h. For accurate mass measurement, leucine enkephalin was used as the lock spray standard at a concentration of 100 ng/mL with a flow rate of 50 μL/min.

Data were processed using MarkerLynx software (Waters). Peak detection was performed across the mass range of m/z 100–1000 with a retention time (RT) between 2 and 12 min, peak widths automatically detected, an intensity threshold of 100, a mass window of 0.02, a RT window of 0.1, and a noise elimination value of 6.0. Multivariate statistical analysis was performed using EZinfo software (Waters) for unsupervised principal component analysis (PCA) to obtain a general overview of the variance of metabolic phenotypes. Next, orthogonal projection to latent structures-discriminant analysis (OPLS-DA) was used to maximize the differences in the metabolic profiles between the wild-type and mutant strains. The metabolite features were identified based on retention behavior, mass assignment, and online database query. Metabolic pathway analysis was performed in the HMDB database (http://www.hmdb.ca/) to investigate the disturbed metabolic pathways and to facilitate biological interpretation.

### Virulence assays of sulfacetamide on cotton

The cotton cotyledon was used for evaluating the virulence of SFA by infiltration. The SFA was dissolved in 2% dimethyl sulfoxide (DMSO) at concentration of 25 mM. The solutions were infiltrated into the cotyledons of 10-day-old cotton seedlings, and the wilting phenotype was investigated after 24 hours; the solvent was used as the control. Virulence assays were also performed on cotton seedlings using the root-dipping method. The bottom of the nutrition pot was wiped out to expose the root of a two-week-old seedling, and it was transferred into a new pot containing only 20.0 mL of at 25 mM SFA solution; the wilting symptoms of the cotton seedling were investigated after 36 hours post inoculation.

## Additional Information

**How to cite this article**: Zhang, D.-D. *et al*. Identification and characterization of a pathogenicity-related gene *VdCYP1* from *Verticillium dahliae*. *Sci. Rep.* **6**, 27979; doi: 10.1038/srep27979 (2016).

## Supplementary Material

Supplementary Information

Supplementary Dataset 1

Supplementary Dataset 2

## Figures and Tables

**Table 1:** Detection of the metabolite variation between the *VdCYP1* deletion strain and the wild-type strain using the UPLC-Q-TOF method.

Description	Matching ID#	WT/Δ*CYP-1*	WT/*CYP-2*
Sulfacetamide	HMDB14772	26.126	39.588
1-Methoxy-1H-indole-3-acetonitrile	HMDB40973	Infinity	1.190
Sinalexin	HMDB32026	19.442	1.248
Penmacric acid	HMDB29436	13.082	1.260
Alloxan	HMDB02818	8.036	1.092
Pomolic acid	HMDB35106	7.610	1.546
Sonchuionoside C	HMDB35212	4.565	1.197
Eszopiclone	HMDB14546	4.000	1.201
Zopiclone	HMDB15329	4.000	1.121
1-(1-Propenylthio)propyl disulfide	HMDB33041	3.945	1.041
(E)-1-Propenyl 1-(propylthio)propyl disulfide	HMDB33072	3.945	2.073
2, 4, 6-Triethyl-1, 3, 5-trithiane	HMDB40262	3.945	1.071
4-Phenylpyridine	HMDB33123	2.571	1.453
Capsiate	HMDB34780	1.953	2.168

^#^The metabolite detection involved searching 3 databases, including the Human Metabolome Database (http://www.hmdb.ca/), the KEGG database (http://www.kegg.jp/kegg/), and the ChEBI database (http://www.ebi.ac.uk/chebi/).
