# Supplementary material for: Identification and characterization of a pathogenicity-related gene VdCYP1 from Verticillium dahliae
Source: Sci Rep. 2016 Jun 22;6:27979. doi: 10.1038/srep27979 (PMC4916405; doi:10.1038/srep27979)
Supplement: Supplementary Information [file srep27979-s1.pdf]

Identification and characterization of a pathogenicity-related gene *VdCYP1* from *Verticillium dahliae*

Dan-Dan Zhang*, Xin-Yan Wang*, Jie-Yin Chen*, Zhi-Qiang Kong, Yue-Jing Gui, Nan-Yang Li, Yu-Ming Bao, Xiao-Feng Dai

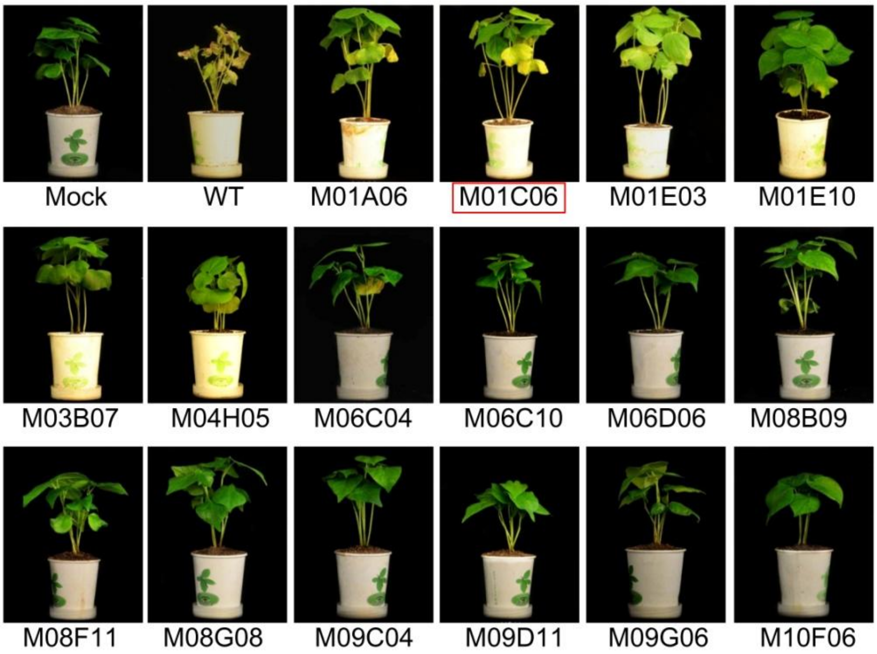

Supplementary Figure 1 | The mutant phenotype of significantly reduced virulence on cotton. The pathogenicity was determined using the root-dipping method with susceptible cotton (cv. Junmian1). WT indicates inoculation with the wild-type strain Vd991, and Mock indicates the control that was treated with sterile water.

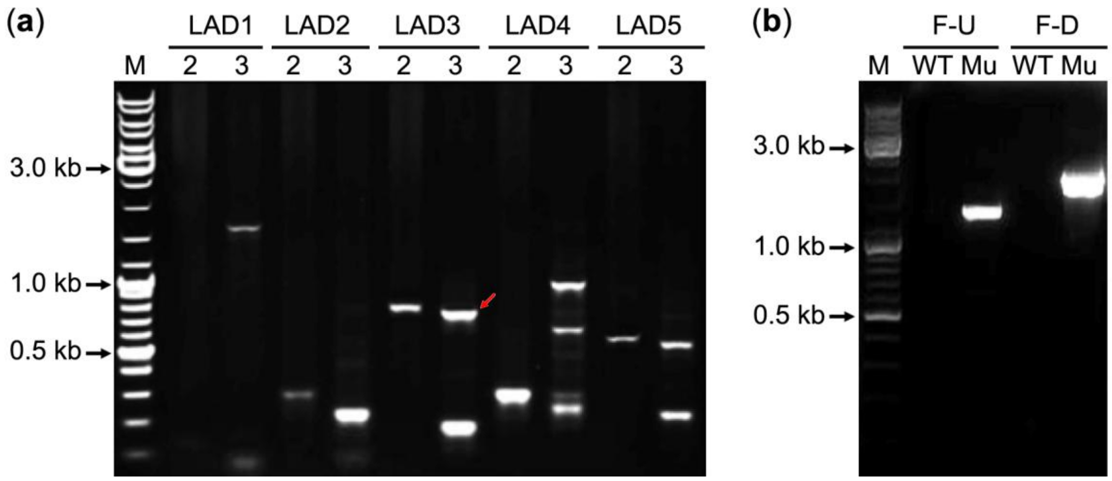

Supplementary Figure 2 | Detection of the genomic integration site of T-DNA in the mutant. (**a**) Detection of the integration site of T-DNA in the mutant genome using the hiTAIL-PCR method. LAD1 - LAD5 represent the 5 degenerate primers (see Supplementary Table S3 online). The amplicon marked with a red arrow represents the flanking fragment of the T-DNA in the mutant, and the numbers '2' and '3' represent the nested PCR from the second and third rounds of the hiTAIL-PCR. (**b**) Validation of the T-DNA insertion using PCR. WT is the wild-type strain Vd991, and Mu represents the mutant. F-U and F-D represent the 2 sequences flanking the T-DNA insertion site.

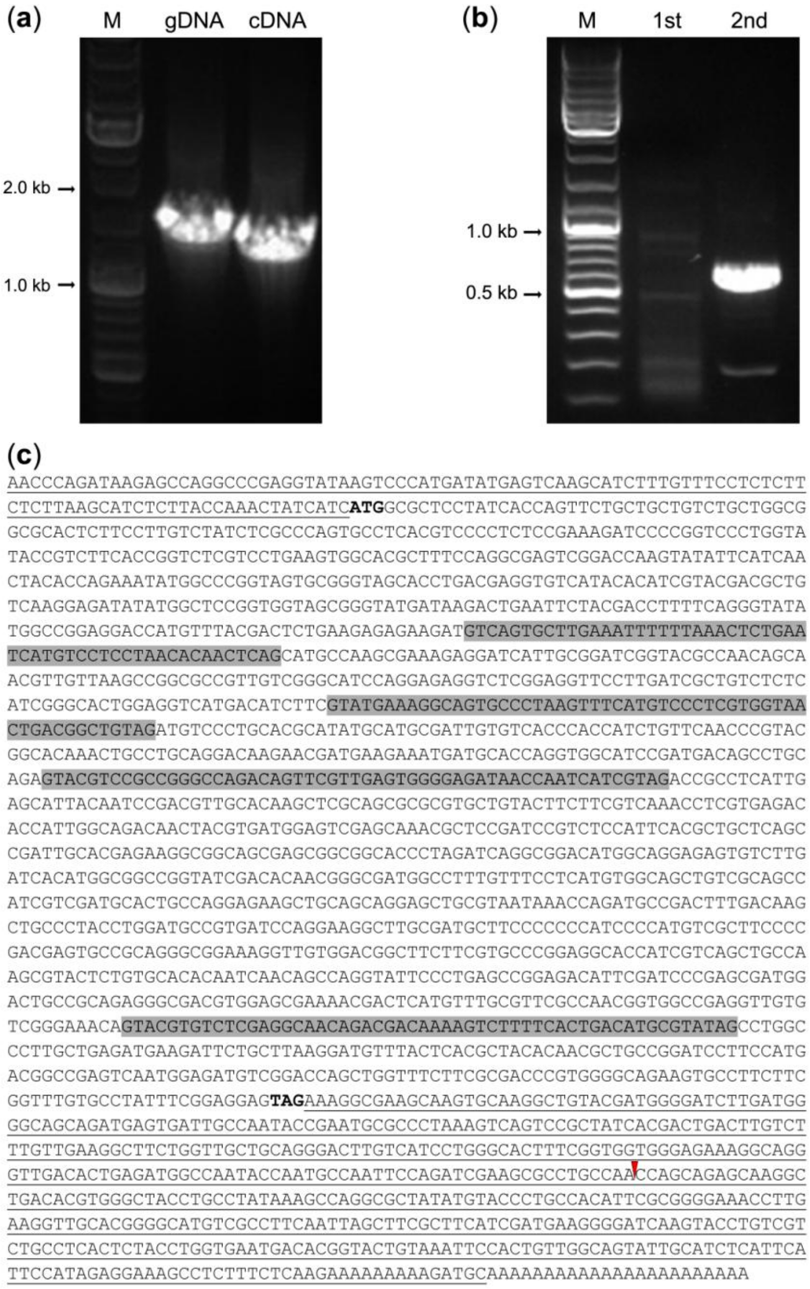

Supplementary Figure 3 | Cloning *VdCYP1* from the Vd991 wild-type strain. (**a**) *VdCYP1* amplification from genomic DNA and cDNA. (**b**) The 3′ UTR was cloned using 3′ RACE (Invitrogen). The first lane represents the first round of amplification with the first-strand cDNA, and the second lane is the nested PCR with the product of the first round. (**c**) The sequence and structure of *VdCYP1*. The start and stop codons are marked in bold type, the introns are colored in a gray shadow, underlined bases represent the 5′ UTR and 3′ UTR, and the red inverted triangle indicates the integration site of the T-DNA in the M01C06 mutant.

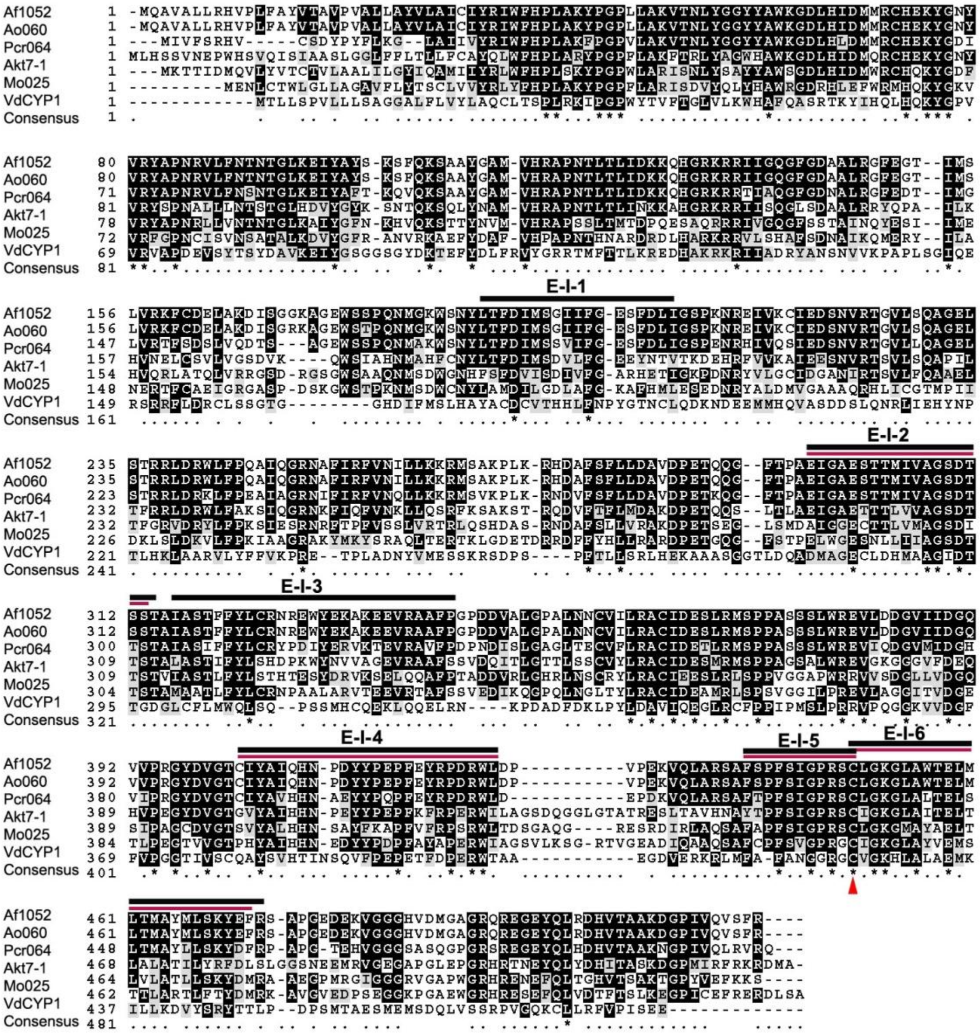

Supplementary Figure 4 | Sequence alignment of *VdCYP1* with its orthologs in other fungi. The sequence alignment was performed using ClustalX and was output with BOXSHADE (online tool, http://mobyle.pasteur.fr/cgi-bin/portal.py). The conserved domain of group I of the E-class P450 was predicted using a InterProScan sequence search (http://www.ebi.ac.uk/Tools/pfa/iprscan/), and the alignment sequence with black lines represents the six conserved motifs (E-I-1 ~ E-I-6). The conserved motifs in VdCYP1 are drawn with red lines, and the conservation of the cysteine heme-iron ligand signature at the C-terminus of the protein is marked with the red triangle. GeneBank ID: Af1052, AFL2G_04850; Ao060, AO090011000045; Pcr064, CAP95980.1; Akt7-1, BAO10618.1; Mo025, MGG_03375.6.

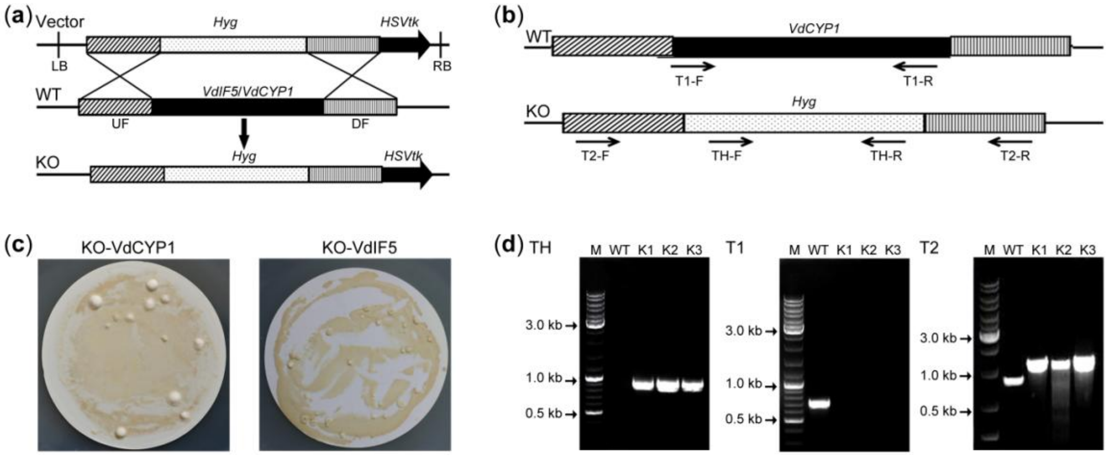

Supplementary Figure 5 | Targeted gene deletion of the two genes of *VdCYP1* and *VdIF5*. (**a**) Schematic diagram of targeted gene deletion by homologous recombination. *Hyg* represents the hygromycin element, UP and DF are the two flanking sequences (~ 1 kb) of targeted deletion gene, and *HSVtk* is the herpes simplex virus thiamine kinase gene (HSVtk) encoded in pGKO2, as a toxic compound for negative selection against ectopic transformants. LB and RB indicate the left and right borders of the T-DNA sequence, respectively. (**b**) Schematic diagram of molecular verification of the targeted gene deletion by PCR. Three primers were designed for PCR validation. The TH primer pairs in the *Hyg* gene can amplify a fragment from the knockout strain but not from the wild-type strain. The T1 primer pairs in the internal *VdCYP1* sequence can amplify a fragment from the wild-type strain but not from the knockout strain. The T2 pairs in the 2 flanking sequences of *VdCYP1* can amplify differently sized fragments in the knockout and wild-type strains. (**c**) Transformants of *VdCYP1* and *VdIF5* deletion. Targeted deletion of *VdIF5* by co-culture with *A. tumefaciens* and the Vd991 wild-type strain was unable to generate transformants. (**d**) Validation of the positive transformants for *VdCYP1* deletion using PCR. The electrophoretograms of TH, T1 and T2 were generated as described (b).

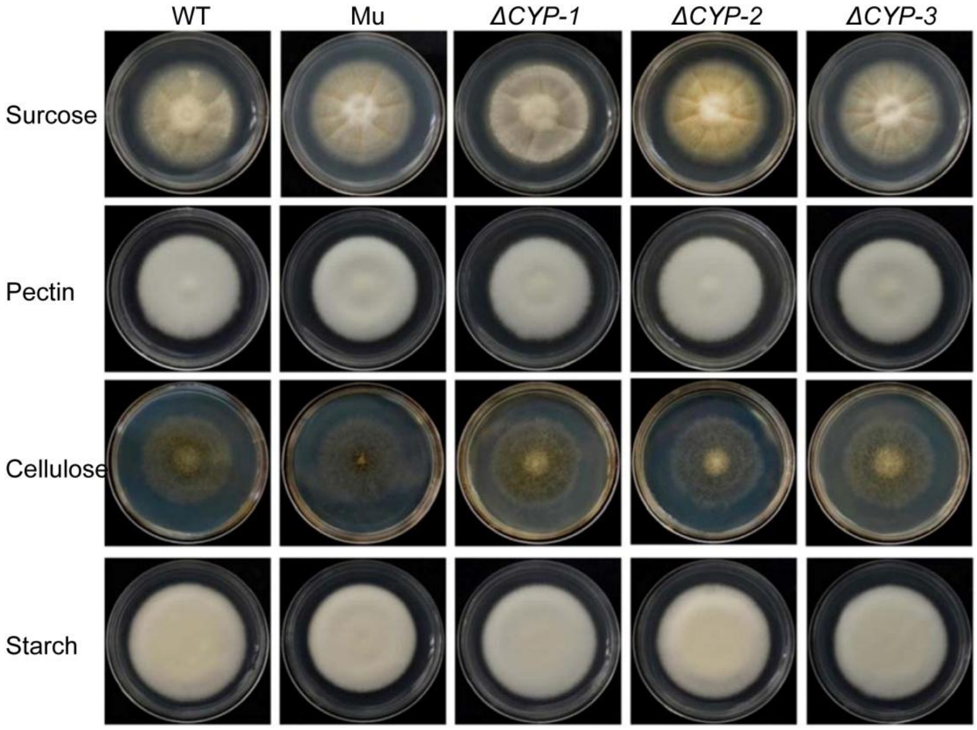

Supplementary Figure 6 | The growth phenotypes of the *VdCYP1* deletion strain cultured on media containing different carbon sources.

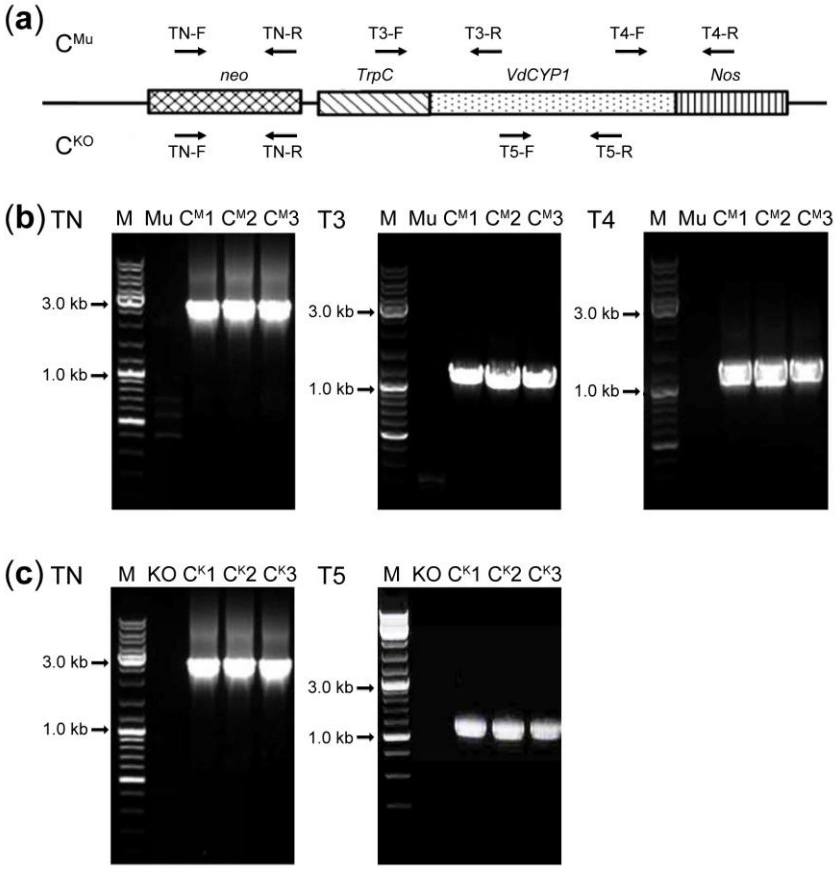

Supplementary Figure 7 | Validation of the complementation transformants using PCR. (**a**) Schematic diagram of the molecular validation of the complementation transformants. Three pairs of primers were designed to detect the re-introduction of *VdCYP1* into the T-DNA mutant (C^{Mu}), including the TN pair for the *neo* gene (encoding aminoglycoside phosphotransferase) and the T3 and T4 pairs for the integration of the *VdCYP1* gene. Two pairs of primers were used to detect the re-introduction of *VdCYP1* into the knockout strain (C^{KO}), namely the TN neo detection primers and the T5 pair for the integration of *VdCYP1* genes. (**b**) Validation of the complementation transformants of the T-DNA mutant using PCR. Mu indicates the M01C06 mutant, and $C^{M}1$, $C^{M}2$, and $C^{M}3$ are the three complementation transformants. (**c**) Validation of the corresponding complementation transformants of the *VdCYP1* deletion strain using PCR. KO indicates the *VdCYP1* deletion strain; $C^{K}1$, $C^{K}2$, and $C^{K}3$ represent the corresponding 3 complementation transformants.

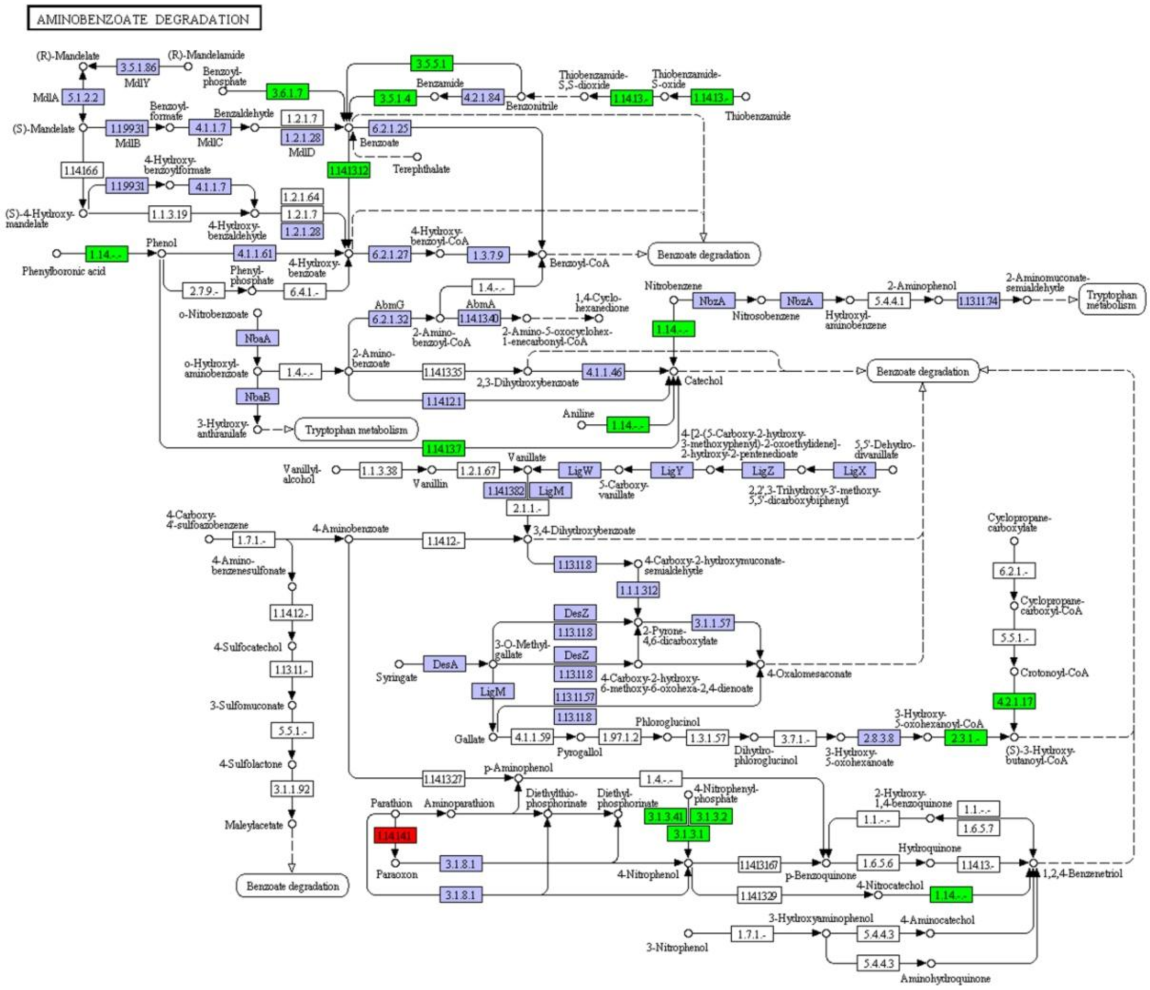

Supplementary Figure 8 | Annotation of the aminobenzoate degradation pathways using the reference genome of VdLs.17. All of the coding genes in VdLs.17 were compared to the KEGG database (http://www.kegg.jp/) using the BLASTP program (e-value$<1e^{-7}$, identities >30%). The genes involved in the aminobenzoate degradation pathway (ko00627) were collected, and the pathway was colored green using KEGG Mapper (http://www.kegg.jp/kegg/tool/map_pathway2.html); the red box represents the matching step of VdCYP1.

Supplementary Table 3

Primer name	Primer sequence (5′-3′)	Description
**T-DNA tagged genes analysis**		
LAD1	ACGATGGACTCCAGAGCGGCCGCVNVNNNGGAA	arbitrary degenerate primer for hiTAIL-PCR
LAD2	ACGATGGACTCCAGAGCGGCCGCBNBNNNGGTT	arbitrary degenerate primer for hiTAIL-PCR
LAD3	ACGATGGACTCCAGAGCGGCCGCVVNVNNNCCAA	arbitrary degenerate primer for hiTAIL-PCR
LAD4	ACGATGGACTCCAGAGCGGCCGCBDNBNNNAGGT	arbitrary degenerate primer for hiTAIL-PCR
LAD5	ACGATGGACTCCAGAGCGGCCGCBHNDNNNGACC	arbitrary degenerate primer for hiTAIL-PCR
RB-1	CGTGACTGGGAAAACCCTGGCGTT	specific primer in T-DNA border for hiTAIL-PCR
RB-2	ACGATGGACTCCAGTCCCCAACTTAATCGCCTTGCAGCACATC	specific primer in T-DNA border for hiTAIL-PCR
RB-3	GAAGAGGCCCGCACCGATCGCCCTT	specific primer in T-DNA border for hiTAIL-PCR
T-UF	GTTGTCGTTGGGTTAGGTTGGC	verify the upstream sequence of the insertion site
T-UR	GGCAGTTCGGTTTCAGGCAGGT	verify the upstream sequence of the insertion site
T-DF	TGACTGGAGCGAGGCGATGTTC	verify the downstream sequence of the insertion site
T-DR	CTGTCGTCTGCCTCACTCTACC	verify the downstream sequence of the insertion site
**VdCYP1 and VdIF1 cloning**		
CYP1-F	CCCCTCAACCCAGATAAGAGCC	*VdCYP1* DNA and cDNA sequences cloning
CYP1- R	TCGTACAGCCTTGCACTTGCTT	*VdCYP1* DNA and cDNA sequences cloning
IF5-F	GCATGGCTCTCGTCAACGTTCGT	*VdIF5* DNA and cDNA sequences cloning
IF5- R	CGCTACTCGTCGTCCGAGTCCTC	*VdIF5* DNA and cDNA sequences cloning
**VdIF5 and VdCYP1 expression detection**		
IF5-QF	CTGGCAGCGACGATGAGTTTGA	RT-qPCR for *VdIF5* gene
IF5-QR	TCCTCATCCTCGTCATCACCAAT	RT-qPCR for *VdIF5* gene
CYP1-QF	ACTACGTGATGGAGTCGAGCAAA	RT-qPCR for *VdCYP1* gene
CYP1-QR	CTGCCACATGAGGAAACAAAGG	RT-qPCR for *VdCYP1* gene
Tubulin-F	TTCCCCCGTCTCCACTTCTTCATG	reference for RT-qPCR

Tubulin-R	TTGCGCATCTGGTCCTCGACCTCCC	reference for RT-qPCR
**Construct VdCYP1 and VdIF5 deletion mutants**		
KO- IF5-UF	TTTCACTGACATGCGTATAGCCT	upstream amplication of gene *VdIF5*
KO- IF5-UR	GCCCAAAAATGCTCCTTCAACCCTTGCCCTCAATCTTGGTCT	upstream amplication of gene *VdIF5*
KO- IF5-DF	CCCTGGGTTCGCAAAGATAACACCTCCAAGAAGGTCCGCAAGG	downstream amplication of gene *VdIF5*
KO- IF5-DR	CAAGGACTTCTGGGACGGGAGC	downstream amplication of gene *VdIF5*
KO- IF5-RF	GGGGACAAGTTTGTACAAAAAAGCAGGCTCCAGGTCGGGCTTTCCACTTCT	nested PCR reaction for final amplification
KO- IF5-LF	GGGGACCACTTTGTACAAGAAAGCTGGGTATTGCGTATTTGCGGCTTTATC	nested PCR reaction for final amplification
KO- CYP1-UF	GATGGGCTCGTAGGCAATGACA	upstream amplication of gene *VdCYP1*
KO- CYP1-UR	GCCCAAAAATGCTCCTTCAAGCAGCAGAACTGGTGATAGGAGC	upstream amplication of gene *VdCYP1*
KO- CYP1-DF	CCCTGGGTTCGCAAAGATAAAAGTGCCTTCTTCGGTTTGTGC	downstream amplication of gene *VdCYP1*
KO- CYP1DR	GGCTACTGGGCTTCCGTTCTAT	downstream amplication of gene *VdCYP1*
KO- CYP1-RF	GGGGACAAGTTTGTACAAAAAAGCAGGCTGGTGCGTTTGTTGATTAGAGGG	nested PCR reaction for final amplification
KO- CYP1-LF	GGGGACCACTTTGTACAAGAAAGCTGGGTTGAAGTTGTCGTTGGGTTAGGTT	nested PCR reaction for final amplification
Hyg-F	TTGAAGGAGCATTTTTGGGC	hygromycin element amplication
Hyg-R	TTATCTTTGCGAACCCAGGG	hygromycin element amplication
CYP1 T1-F	GGATCGGTACGCCAACAGCAAC	verify the positive transformants of *VdCYP1* delection mutant
CYP1 T1-R	GCTTGTCAAAGTCGGCATCTGG	verify the positive transformants of *VdCYP1* delection mutant
CYP1 T2-F	TCTTTCCGTTACCTAACAATTATCCC	verify the positive transformants of *VdCYP1* delection mutant
CYP1 T2-R	GCACTTGCTTCGCCTTTCTACTC	verify the positive transformants of *VdCYP1* delection mutant
CYP1 TH-F	GCAGACAGGAACGAGGACAT	verify the positive transformants of hygromycin element
CYP1 TH-R	GCTCCATACAAGCCAACCAC	verify the positive transformants of hygromycin element

**Complementary to VdCYP1 deletion mutant**		
C-Trp-F	TCCTACCTTCCAATCGATACCG	*TrpC*-promoter sequence amplication
C-Trp-R	TACGTAAAGAATTGGGAATTTCATTTGGATGCTTGGGTAGAATAGGTA	*TrpC*-promoter sequence amplication
C-CYP1-F	ATGGCGCTCCTATCACCAGTTC	*VdCYP1* cDNA amplication
C-CYP1-R	CTACTCCTCCGAAATAGGCACA	*VdCYP1* cDNA amplication
C-Nos-F	GGATTCACAAGCTAGTTCCCTAAATCCACTTAACGTTACTGAAATC	*Nos*-terminator fragment amplication
C-Nos-R	AACATGCAATTATCTTTGCG	*Nos*-terminator fragment amplication
CF1-F	GGTACCATGGCGCTCCTATCACCAGTTC	nested PCR reaction for *TrpC*, *VdCYP1* and *Nos* fragments
CF1-R	TCTAGACTACTCCTCCGAAATAGGCACA	nested PCR reaction for *TrpC*, *VdCYP1* and *Nos* fragments
TN-F	GTTTGCGGGCTGTCTTGACG	*neo* amplication in the positive complementation transformants
TN-R	TACCTGTGCATTCTGGGTAA	*neo* amplication in the positive complementation transformants
T3-F	GCCCTTCCTCCCTTTATTTCAG	verify the upstream integration of *VdCYP1* gene in positive complementation transformants of mutant M01C06
T3-R	GTCGGCATCTGGTTTATTACGC	verify the upstream integration of *VdCYP1* gene in positive complementation transformants of mutant M01C06
T4-F	GTGTCACCCACCATCTGTTCAA	verify the downstream integration of *VdCYP1* gene in positive complementation transformants of mutant M01C06
T4-R	GATCTGCTTGATCTCGTCTCCC	verify the downstream integration of *VdCYP1* gene in positive complementation transformants of mutant M01C06
T5-F	GGATCGGTACGCCAACAGCAAC	verify the integration of *VdCYP1* gene in positive complementation transformants of the *VdCYP1* deletion mutant
T5-R	GCTTGTCAAAGTCGGCATCTGG	verify the integration of *VdCYP1* gene in positive complementation transformants of the *VdCYP1* deletion mutant
